# Gut microbial characteristics and kynurenine-related metabolism across sarcopenia groups: associations with neuromuscular and physical function

**DOI:** 10.3389/fnagi.2026.1883616

**Published:** 2026-09-25

**Authors:** Chang Fang, Chen Wang, Rui Wang, Yi-fan Lu, Ling Ma

**Affiliations:** 1 School of Sports Medicine and Rehabilitation, Beijing Sport University, Beijing, China; 2 College of Sports and Exercise Rehabilitation, Jinzhou Medical University, Jinzhou, Liaoning, China; 3 School of Medical Humanities, Anhui Medical University, Hefei, Anhui, China

**Keywords:** C-terminal agrin fragment, gut microbiota, kynurenine, neuromuscular junction, physical performance, sarcopenia, tryptophan metabolism

## Abstract

**Background:**

Sarcopenia is characterized by heterogeneous alterations in muscle mass, strength, and physical performance and may involve interactions among the gut microbiota, tryptophan metabolism, and neuromuscular function. However, these biological characteristics across different sarcopenia stages remain incompletely understood. This study investigated gut microbial characteristics, kynurenine-related metabolism, circulating biomarkers, and physical function in older adults with different sarcopenia status.

**Methods:**

In this cross-sectional study, 67 older adults were classified according to the Asian Working Group for Sarcopenia 2019 criteria as non-sarcopenia (NSG, *n* = 17), possible sarcopenia (PSG, *n* = 20), or sarcopenia (SG, *n* = 30). Muscle mass, handgrip strength, and multidimensional physical performance were assessed. Serum C-terminal agrin fragment (CAF), tumor necrosis factor-α (TNF-α), amino acids, kynurenine (KYN), and tryptophan (Trp) were measured, and the KYN/Trp ratio was calculated. Gut microbiome composition was characterized by shotgun metagenomic sequencing. Differential abundance was evaluated using MaAsLin2 with adjustment for age and body mass index (BMI), and exploratory correlations among microbial genera, metabolites, circulating biomarkers, and physical function were examined with false discovery rate correction.

**Results:**

ASMI was significantly lower in the SG group than in the NSG and PSG groups, whereas several measures of physical performance differed among groups without a uniform stepwise pattern. No significant differences were observed in alpha diversity, beta diversity, or the Firmicutes/Bacteroidetes ratio, and sarcopenia group explained only 3% of the variation in overall microbial community composition (PERMANOVA, *R*^2^ = 0.03, *p* = 0.226). Several genus-level associations were nominally significant after adjustment for age and BMI, but none remained significant after BH-FDR correction. After adjustment for age and BMI, serum KYN concentrations and the KYN/Trp ratio were significantly higher in the SG group than in the NSG group (*p* = 0.011 and *p* = 0.043, respectively), whereas Trp and the measured amino acids showed no significant pairwise differences. The unadjusted increase in CAF across sarcopenia groups was attenuated after covariate adjustment, and TNF-α did not differ significantly among groups. Exploratory correlation analyses identified several FDR-significant associations across microbial, metabolic, and functional domains, including negative associations of KYN and KYN/Trp with handgrip strength and a positive association between CAF and 6-m gait speed(GS).

**Conclusion:**

Established sarcopenia was associated with altered circulating kynurenine-related metabolism despite the absence of pronounced differences in overall gut microbial community structure. Exploratory cross-domain associations further suggest potential interactions among gut microbial characteristics, tryptophan metabolism, and neuromuscular and physical function. These findings support further investigation of host–microbial tryptophan metabolism in sarcopenia but do not establish a causal microbiota–kynurenine–NMJ pathway.

## 1 Introduction

With the acceleration of global population aging, sarcopenia has become a major health concern in older adults. It is characterized by declines in skeletal muscle mass, strength, and physical performance and is associated with increased risks of falls, disability, hospitalization, and mortality (Shim et al., 2025). Although earlier definitions emphasized low muscle mass (Chen et al., 2020; Cruz-Jentoft et al., 2010), muscle strength and physical performance are increasingly recognized as important components of sarcopenia. Accordingly, the Asian Working Group for Sarcopenia (AWGS 2019) introduced “possible sarcopenia” to facilitate early identification of older adults with reduced muscle strength and/or physical performance before confirmation of low muscle mass (Cao et al., 2022; Lim et al., 2022). However, the biological characteristics associated with different sarcopenia stages remain incompletely understood.

The neuromuscular junction (NMJ) is essential for signal transmission between motor neurons and skeletal muscle fibers and undergoes structural and functional remodeling with aging, potentially contributing to denervation and muscle dysfunction (Gonzalez-Freire et al., 2014; Piasecki et al., 2016; Pratt et al., 2021). Maintenance of NMJ integrity depends on the agrin–low-density lipoprotein receptor-related protein 4 (LRP4)–muscle-specific receptor tyrosine kinase (MuSK) signaling pathway, in which motor neuron-derived agrin interacts with LRP4 to activate MuSK signaling and promote acetylcholine receptor clustering required for effective neuromuscular transmission. Disruption of this pathway has been implicated in impaired NMJ stability and age-related neuromuscular dysfunction. C-terminal agrin fragment (CAF), a circulating product of agrin cleavage, has been proposed as a biomarker associated with NMJ remodeling, and elevated CAF concentrations have been linked to reduced muscle strength and sarcopenia-related phenotypes in older adults (Landi et al., 2016; Marzetti et al., 2014; Pratt et al., 2021). However, its relationship with different sarcopenia stages remains insufficiently characterized.

Sarcopenia also develops within the context of age-related metabolic and inflammatory changes (Antuña et al., 2022; Dalle et al., 2017). The emerging gut–muscle axis highlights potential interactions among the gut microbiota, microbial metabolites, host immune regulation, and skeletal muscle metabolism (Bao et al., 2020; Ticinesi et al., 2017). Tryptophan (Trp) metabolism is particularly relevant because it involves both host and microbial pathways (Xu et al., 2024). Host tissues metabolize Trp through the KYN pathway, which is influenced by inflammatory signaling and generates metabolites with diverse neuroactive and metabolic properties (Chung et al., 2023; Westbrook et al., 2020). However, how gut microbial characteristics, Trp-related metabolism, inflammatory status, NMJ-related biomarkers, and physical function are associated across sarcopenia stages remains poorly understood.

The novelty of the present study lies in integrating clinical phenotypes, neuromuscular biomarkers, gut microbial characteristics, and tryptophan-related metabolic alterations across the continuum from non-sarcopenia to possible sarcopenia and established sarcopenia. Rather than establishing a causal mechanism, this study provides an exploratory framework linking microbial, metabolic, neuromuscular, and functional domains associated with sarcopenia progression.

## 2 Experimental procedures

### 2.1 Study design and participants

This cross-sectional study recruited volunteers from a nursing care center in Langfang, Hebei Province, China. The inclusion criteria were as follows: (1) age ≥65 years; (2) normal communication ability; (3) ability to complete physical performance tests; (4) percentage body fat (PBF) ≤ 29.7% for men and ≤37.2% for women; and (5) provision of written informed consent.

The exclusion criteria were: (1) individuals with specific safety risks, including implanted stents or pacemakers, history of joint replacement, or fractures within the past 3 months; (2) use of antibiotics or probiotic preparations within 4 weeks prior to sampling; (3) history of diarrhea or other gastrointestinal disorders within the past 4 weeks; (4) diabetes with severe complications; and (5) other serious diseases, such as malignancy, chronic kidney disease, severe hepatic or renal dysfunction, asthma, chronic heart failure, severe depression, or other psychiatric disorders.

Participants were classified according to the 2019 consensus of the Asian Working Group for Sarcopenia (AWGS 2019) (Chen et al., 2020). Diagnostic criteria included: (1) Low muscle mass (LMM): Appendicular skeletal muscle mass index (ASMI), calculated as appendicular skeletal muscle mass (ASM) divided by height squared (kg/m^2^). ASMI was measured by multi-frequency bioelectrical impedance analysis, with cut-off values of <7.0 kg/m^2^ for men and <5.7 kg/m^2^ for women. (2) Low muscle strength (LMS): Handgrip strength (HGS), with diagnostic thresholds of <28 kg for men and <18 kg for women. (3) Low physical performance (LPP): 6-m gait speed(GS) < 1.0 m/s, or five-repetition chair stand test (CS-5) time ≥12 s, or Short Physical Performance Battery (SPPB) score ≤9.

Sarcopenia was defined as the presence of LMM plus LMS or LMM plus LPP. Possible sarcopenia was defined as LMS and/or LPP without confirmed LMM. Severe sarcopenia was defined as the simultaneous presence of LMM, LMS, and LPP.

### 2.2 Sample size and statistical considerations

Because this study was designed as an exploratory cross-sectional investigation integrating clinical, biochemical, metabolomic, and microbiome data, the sample size was determined based on feasibility and available recruitment rather than *a priori* power calculation. A sensitivity analysis was subsequently conducted to characterize the detectable effect size under the available sample size. With 67 participants across three groups, a two-sided α level of 0.05, and 80% statistical power, the study could detect an overall between-group effect of approximately Cohen’s *f* = 0.39. Therefore, the available sample size was sufficient to detect moderate-to-large overall group effects, while smaller effects, particularly those involving high-dimensional microbiome features, may not have been detectable.

A total of 103 participants who met the inclusion and exclusion criteria were initially enrolled and completed physical performance assessments. During subsequent sample collection and laboratory analyses, 18 participants were excluded due to unavailable blood samples, 9 participants were excluded due to unavailable fecal samples for microbiome analysis, and 9 participants were excluded because their biochemical measurements did not meet predefined laboratory quality control criteria. Finally, 67 participants with complete clinical, biochemical, metabolomic, and microbiome datasets were included in the final analysis (Figure 1).

**Figure 1:**
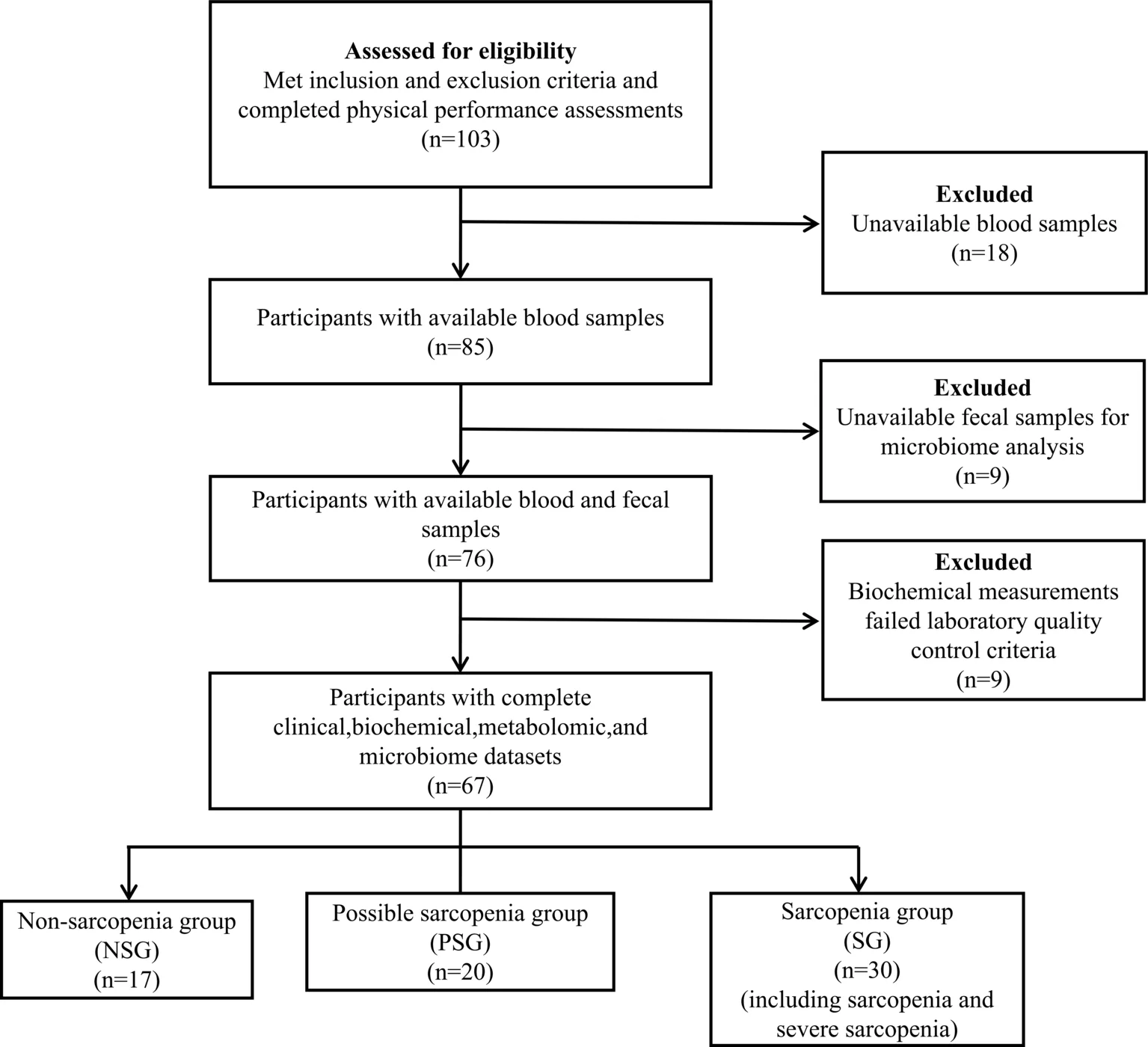
Flow chart of participant enrollment.

Based on the AWGS 2019 diagnostic criteria, participants were categorized into three groups: the non-sarcopenia group (NSG; *n* = 17), possible sarcopenia group (PSG; *n* = 20), and sarcopenia group (SG; *n* = 30). Participants meeting the criteria for sarcopenia or severe sarcopenia were combined into the SG group because of the relatively small number of individuals with severe sarcopenia.

### 2.3 Sample collection

#### 2.3.1 Blood collection and storage

Fasting venous blood samples (5 mL) were collected from the antecubital vein in the morning. Blood samples were allowed to clot at room temperature for 30 min and subsequently centrifuged at 4 °C and 3,000 rpm for 5 min. The resulting serum was aliquoted and immediately stored at −80 °C until analysis of amino acids, tumor necrosis factor-α (TNF-α), and CAF.

#### 2.3.2 Fecal sample collection and storage

Participants collected the first fecal sample of the day using a standardized collection kit containing a stabilization reagent. Written instructions were provided to ensure standardized sampling procedures. Approximately 0.5 g of fecal material was collected and immediately transferred into preservation tubes according to the manufacturer’s instructions. Samples were transported and stored under recommended conditions until shotgun metagenomic sequencing.

### 2.4 Serum inflammatory cytokines and CAF

Serum concentrations of TNF-α and CAF were measured using commercially available enzyme-linked immunosorbent assay (ELISA) kits (Jianglai Biotechnology, Shanghai, China) according to the manufacturer’s instructions.

### 2.5 Gut microbiome metagenomic sequencing and bioinformatic analysis

Fecal DNA extraction, sequencing library preparation, and shotgun metagenomic sequencing were performed by BGI Genomics (Shenzhen, China).

DNA extraction. Fecal samples were thoroughly homogenized prior to DNA extraction. Approximately 0.15–0.20 mL of each sample was transferred to a 2.0-mL tube containing 500 mg of glass beads (0.1–0.6 mm) and two steel beads. Samples were lysed using Buffer ATL/PVP-10 and Buffer PCI, followed by high-speed bead beating and incubation at 65 °C for 20 min. After centrifugation at ≥14,000 × *g* for 10 min, the supernatant was collected and treated with RNase A. DNA was subsequently purified using magnetic particles and Proteinase K, followed by washing and elution. DNA concentration, integrity, and purity were assessed using fluorescence-based quantification, spectrophotometry, and 1% agarose gel electrophoresis.

Library preparation and sequencing. For library preparation, 1 μg of genomic DNA was fragmented using a *Covaris ultrasonicator*, and fragments of approximately 200–400 bp were selected using magnetic beads. Fragmented DNA underwent end repair, dA-tailing, and adapter ligation using MGIEasy DNA adapters. The adapter-ligated DNA was subsequently amplified by PCR and purified using DNA Clean Beads. PCR products were subjected to single-strand circularization and enzymatic digestion, followed by purification. Qualified circularized libraries were converted into DNA nanoballs (DNBs) by rolling-circle amplification and sequenced on the DNBSEQ platform (BGI Genomics, Shenzhen, China).

Sequencing data quality control. Raw sequencing reads were subjected to quality filtering before downstream analysis. Reads were removed if (1) more than 10% of bases were ambiguous (N); (2) they contained adapter contamination with an overlap of ≥15 bp; or (3) more than 40% of bases had a Phred quality score below Q20. Host-derived sequences were additionally removed before downstream microbial taxonomic analyses.

### 2.6 Targeted metabolomic analysis

Targeted metabolomic analysis was performed using ultra-performance liquid chromatography–tandem mass spectrometry (UPLC–MS/MS). Serum amino acids and tryptophan-related metabolites, including Trp and KYN, were quantified in multiple reaction monitoring (MRM) mode using a QTRAP 5500 LC–MS/MS system (SCIEX, Framingham, MA, USA) coupled with a Waters UPLC system (Waters Corporation, Milford, MA, USA). Data were processed and quantified using MultiQuant software (SCIEX, USA). The KYN/Trp ratio was calculated to characterize the relative balance between circulating KYN and Trp.

### 2.7 Assessment of muscle mass, strength, and physical performance

Appendicular skeletal muscle mass was measured in the standing position using the InBody S10 (InBody Co., Seoul, Korea). ASMI was calculated as appendicular muscle mass divided by height squared (m^2^) (Baumgartner et al., 1998).

Physical performance assessments included handgrip strength (HGS) (Zhang et al., 2021), 6-min walk test (6MWT) (Gao et al., 2021), arm curl test (Bennell et al., 2011), 30-s chair stand test (CST), Timed Up and Go Test (TUGT) (Bennell et al., 2011), SPPB, and the Berg Balance Scale. Before testing, investigators explained and demonstrated each procedure. To minimize fatigue, upper- and lower-limb tests were alternated with approximately 3-min intervals between tests.

For the HGS test (EH101 dynamometer), participants stood with feet shoulder-width apart, arms extended downward, and elbows in a neutral position (index finger flexed at approximately 90°). Participants were instructed to exert maximal grip strength for 3 s, with 60-s rest intervals between trials.

For the 6-m walking test, the time required to complete the 6-m walking distance was recorded in seconds. For the 6-min walk test (6MWT), participants walked along a 50-m rectangular track for 6 min, and the total distance covered was recorded in meters.

The TUGT assessed dynamic balance: participants rose from a seated position, walked to a cone placed 3 m away, turned, and returned to sit down as quickly as possible.

The CST and arm curl test assessed lower- and upper-limb strength, respectively. Participants performed as many sit-to-stand repetitions as possible within 30 s (CST) and completed as many arm curls as possible within 30 s (8-lb dumbbell for men and 5-lb dumbbell for women) (Milanović et al., 2013).

### 2.8 Statistical analysis

All statistical analyses were performed using R software (version 4.4.1). Continuous variables were assessed for normality using the Shapiro–Wilk test together with visual inspection of Q–Q plots, and homogeneity of variance was evaluated using Levene’s test. Normally distributed continuous variables are presented as mean ± standard deviation (SD), whereas non-normally distributed variables are presented as median [interquartile range (IQR)]. Categorical variables are presented as number and percentage.

For comparisons among the NSG, PSG, and SG groups, normally distributed continuous variables with homogeneous variances were analyzed using one-way analysis of variance (ANOVA), followed by Tukey’s honestly significant difference test when the overall group effect was significant. Normally distributed variables with unequal variances were analyzed using Welch’s ANOVA followed by the Games–Howell test. Non-normally distributed variables were analyzed using the Kruskal–Wallis test, followed by Dunn’s test with adjustment for multiple pairwise comparisons when appropriate. Categorical variables were compared using the chi-square test or Fisher’s exact test, as appropriate. Effect sizes were reported as *η*^2^ for one-way ANOVA, *ε*^2^ for Kruskal–Wallis tests, and Cramér’s V for categorical comparisons, where applicable.

For circulating CAF and TNF-α, unadjusted between-group comparisons were performed as described above. Given the between-group differences in participant characteristics, additional multivariable linear regression models were conducted as sensitivity analyses, with sarcopenia group as the primary explanatory variable and age and body mass index (BMI) included as covariates. Adjusted group estimates, 95% confidence intervals (CIs), effect sizes, and *p* values were reported in the Supplementary material.

For gut microbiome analyses, alpha diversity was assessed using the Richness, Shannon, and Simpson indices. Differences in alpha-diversity indices among the three groups were evaluated using the Kruskal–Wallis test. Beta diversity was assessed using principal coordinates analysis (PCoA) based on Bray–Curtis dissimilarities, and differences in overall microbial community composition among groups were evaluated using permutational multivariate analysis of variance (PERMANOVA). Taxonomic relative abundance profiles at the phylum and genus levels were used for descriptive characterization of microbial composition.

Genus-level differential abundance was evaluated using MaAsLin2, with microbial relative abundance as the dependent variable, sarcopenia group as the primary explanatory variable, and age and BMI included as covariates. Pairwise comparisons were performed among the NSG, PSG, and SG groups. Regression coefficients (*β*), standard errors (SEs), raw *p* values, and Benjamini–Hochberg false discovery rate (BH-FDR)-adjusted *q* values were reported. A *q* value < 0.05 was considered statistically significant after correction for multiple testing. Taxa with nominal *p* < 0.05 but *q* ≥ 0.05 were regarded as exploratory associations and interpreted cautiously.

Between-group differences in targeted amino acids and kynurenine-related metabolites were evaluated using linear regression models with group as the primary explanatory variable and age and BMI as covariates. Estimated marginal means and pairwise contrasts among the NSG, PSG, and SG groups were obtained using the *emmeans* package. Adjusted mean differences, 95% CIs, and two-sided *p* values were reported. These analyses were considered prespecified comparisons of targeted metabolites, and statistical significance was defined as *p* < 0.05.

Pearson correlation analyses were performed to explore associations among candidate microbial genera, kynurenine-related metabolites, circulating biomarkers, and muscle and physical performance measures. Candidate genera were selected based on nominal associations (*p* < 0.05) identified in the covariate-adjusted MaAsLin2 analyses and were therefore considered exploratory. Correlation coefficients (*r*) and corresponding *p* values were calculated using individual-level observed measurements. To account for multiple correlation tests, *p* values were adjusted using the Benjamini–Hochberg FDR procedure, with *q* < 0.05 considered statistically significant. All statistical tests were two-sided.

## 3 Results

### 3.1 Baseline characteristics of the study participants

The baseline characteristics of the participants are presented in Table 1. No statistically significant difference in sex distribution was observed among the three groups (*p* = 0.080). Significant between-group differences were observed for age (*p* = 0.001) and BMI (*p* < 0.001).

**Table 1:** Baseline characteristics of participants across the three sarcopenia groups.

Characteristic	NSG (*n* = 17)	PSG (*n* = 20)	SG (*n* = 30)	*p*-value
Sex, *n* (%)				0.080
Female	15 (88.2%)	14 (70.0%)	17 (56.7%)	
Male	2 (11.8%)	6 (30.0%)	13 (43.3%)	
Age, years	78.2 (5.65)	83.1 (4.68)	84.5 (5.74)	0.001
BMI, kg/m^2^	23.9 (2.65)	26.4 (3.77)	22.0 (2.21)	<0.001

Data are presented as mean (SD) or *n* (%), as appropriate. Between-group differences in continuous variables were assessed using one-way analysis of variance (ANOVA), whereas categorical variables were compared using the chi-square test or Fisher’s exact test, as appropriate. BMI, body mass index; NSG, non-sarcopenia group; PSG, possible sarcopenia group; SG, sarcopenia group.

### 3.2 Differences in muscle mass, handgrip strength, and physical performance among the three groups

As shown in Table 2, significant between-group differences were observed in ASMI(*p* < 0.001). *Post hoc* comparisons showed that ASMI was significantly lower in the SG group than in both the NSG (*p* = 0.002) and PSG (*p* < 0.001) groups, whereas no significant difference was observed between the NSG and PSG groups (*p* = 0.950). No significant overall difference in HGS was observed among the three groups (*p* = 0.107).

**Table 2:** Muscle mass, handgrip strength, and physical performance across the three sarcopenia groups.

Variable	NSG (*n* = 17)	PSG (*n* = 20)	SG (*n* = 30)	Overall *P*
ASMI, kg/m^2^	6.66 (0.85)ᵃ	6.74 (0.67)ᵃ	5.79 (0.85)ᵇ	<0.001
HGS, kg	24.5 (4.42)	21.9 (6.17)	20.6 (6.66)	0.107
6-m gait speed, s	2.10 [1.98–2.39]ᵃ	2.83 [2.60–3.15]ᵇ	3.26 [2.90–3.65]ᵇ	<0.001
TUGT, s	5.88 [5.46–6.82]ᵃ	8.52 [6.99–10.2]ᵇ	7.39 [5.92–8.26]ᶜ	<0.001
CS-5, s	8.10 [7.42–9.92]ᵃ	10.6 [8.54–13.5]ᵃᵇ	10.1 [8.63–12.1]ᵇ	0.043
6MWT, m	461 (127)ᵃ	304 (103)ᵇ	380 (85.4)ᶜ	<0.001
ACT, repetitions	19.4 (3.24)ᵃ	16.4 (3.70)ᵃᵇ	15.7 (4.59)ᵇ	0.012
CST, repetitions	17.6 (3.04)ᵃ	12.4 (4.27)ᵇ	15.3 (4.81)ᵃᵇ	0.002
SPPB, points	12.0 [11.0–12.0]ᵃ	9.00 [7.00–11.0]ᵇ	10.0 [8.25–11.0]ᵇ	<0.001
Berg, points	55.0 [54.0–56.0]ᵃ	52.5 [45.8–56.0]ᵃᵇ	51.5 [47.5–55.0]ᵇ	0.012

Data are presented as mean (SD) for normally distributed variables and median [interquartile range (IQR)] for non-normally distributed variables. Overall, between-group differences were assessed using one-way analysis of variance (ANOVA) for normally distributed variables and the Kruskal–Wallis test for non-normally distributed variables. Post hoc pairwise comparisons were performed using Tukey’s test following ANOVA and Wilcoxon rank-sum tests with Bonferroni correction following the Kruskal–Wallis test. Values within the same row that do not share a superscript letter (a, b, or c) are significantly different at *p* < 0.05.

Significant overall between-group differences were also observed in GS, TUGT, CS-5, 6MWT, ACT, CST, SPPB, and Berg Balance Scale scores (all *p* < 0.05). *Post hoc* analyses showed that the NSG group generally exhibited better physical performance than the PSG and/or SG groups across multiple functional measures. However, the between-group differences did not follow a uniform stepwise pattern across all tests. Notably, the PSG group performed significantly worse than the SG group in the TUGT and 6MWT.

### 3.3 Intergroup differences in TNF-α and the neuromuscular junction degeneration marker CAF

As shown in Figure 2, serum CAF levels tended to increase across the NSG, PSG, and SG groups. In the unadjusted analysis, CAF levels were significantly higher in the SG group than in the NSG group (*p* < 0.05), whereas no significant differences were observed between the NSG and PSG groups or between the PSG and SG groups. No significant between-group differences were observed in TNF-α levels.

**Figure 2:**
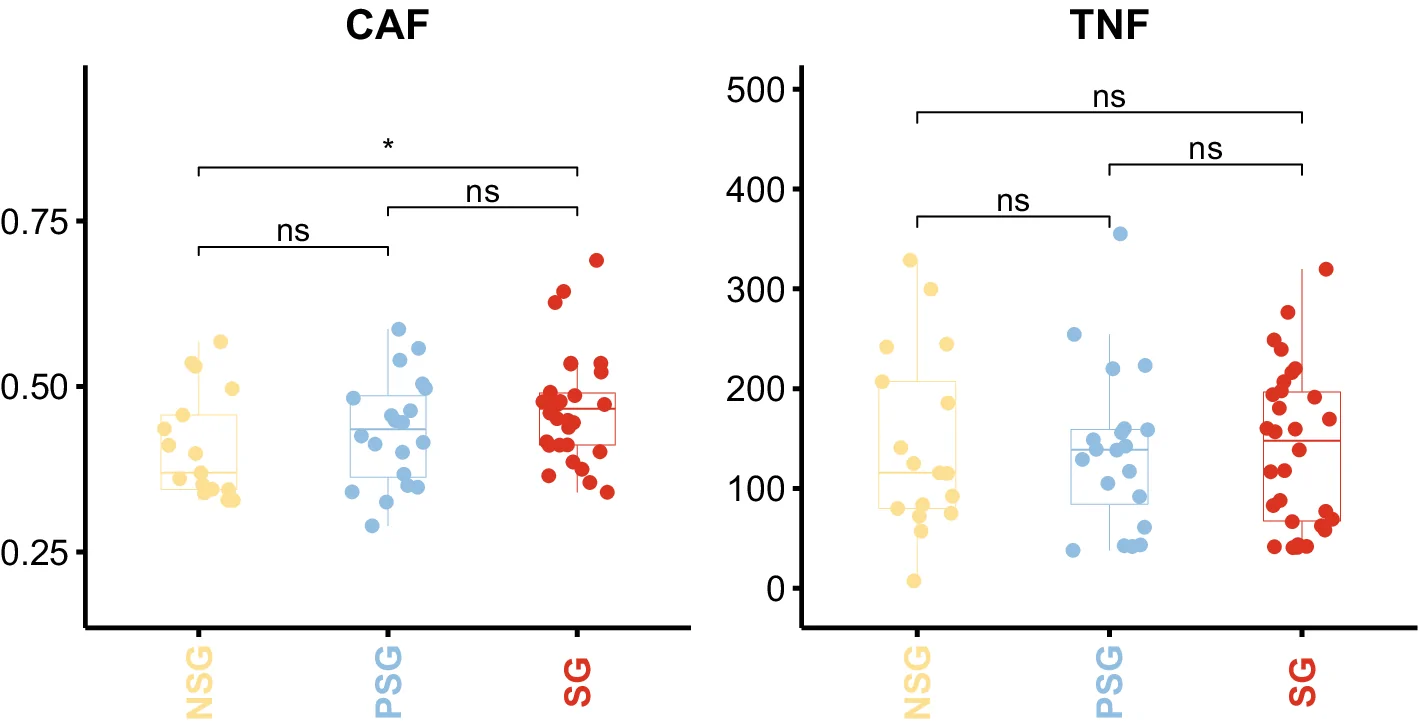
Serum CAF and TNF-α levels across the three sarcopenia groups. Individual data points represent individual participants. Pairwise comparisons shown in the figure are based on unadjusted analyses. *p* < 0.05 was considered statistically significant. *p* < 0.05; ns, not significant. CAF, C-terminal agrin fragment; TNF-α, tumor necrosis factor-α; NSG, non-sarcopenia group; PSG, possible sarcopenia group; SG, sarcopenia group.

Given the significant differences in age and BMI among the three groups, additional multivariable analyses adjusting for age and BMI were performed as sensitivity analyses. After adjustment, the between-group differences in CAF were attenuated and no longer reached statistical significance, while no significant adjusted group differences were observed for TNF-α. Detailed adjusted estimates, 95% confidence intervals, effect sizes, and *p* values are provided in Supplementary Table S1.

### 3.4 Gut microbiome characteristics across the three groups

#### 3.4.1 Species accumulation curve

The species accumulation curve is shown in Figure 3. The x-axis represents the number of samples, and the y-axis represents the cumulative number of microbial species detected. As the number of samples increased, the number of detected species gradually increased, with the curve tending toward a plateau at higher sample numbers. This pattern suggests that additional sampling yielded progressively fewer newly detected species and that the available samples captured a substantial proportion of the detectable microbial species in the study population.

**Figure 3:**
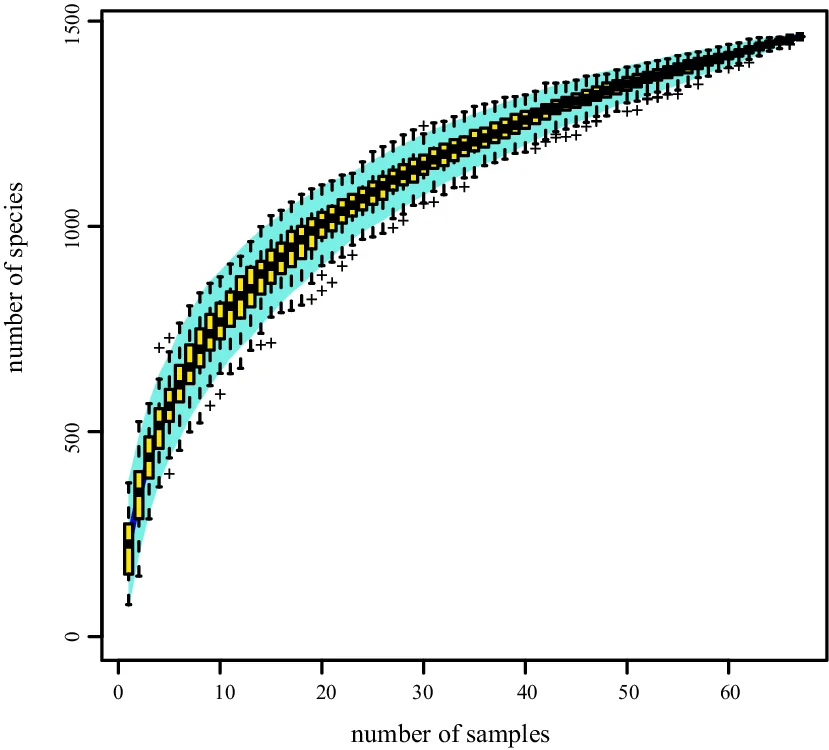
Species accumulation curve.

#### 3.4.2 Alpha and beta diversity analyses

Alpha diversity was assessed using the Richness, Simpson, and Shannon indices. As shown in Figure 4, no significant differences in these alpha-diversity indices were observed among the NSG, PSG, and SG groups (all *p* > 0.05).

**Figure 4:**
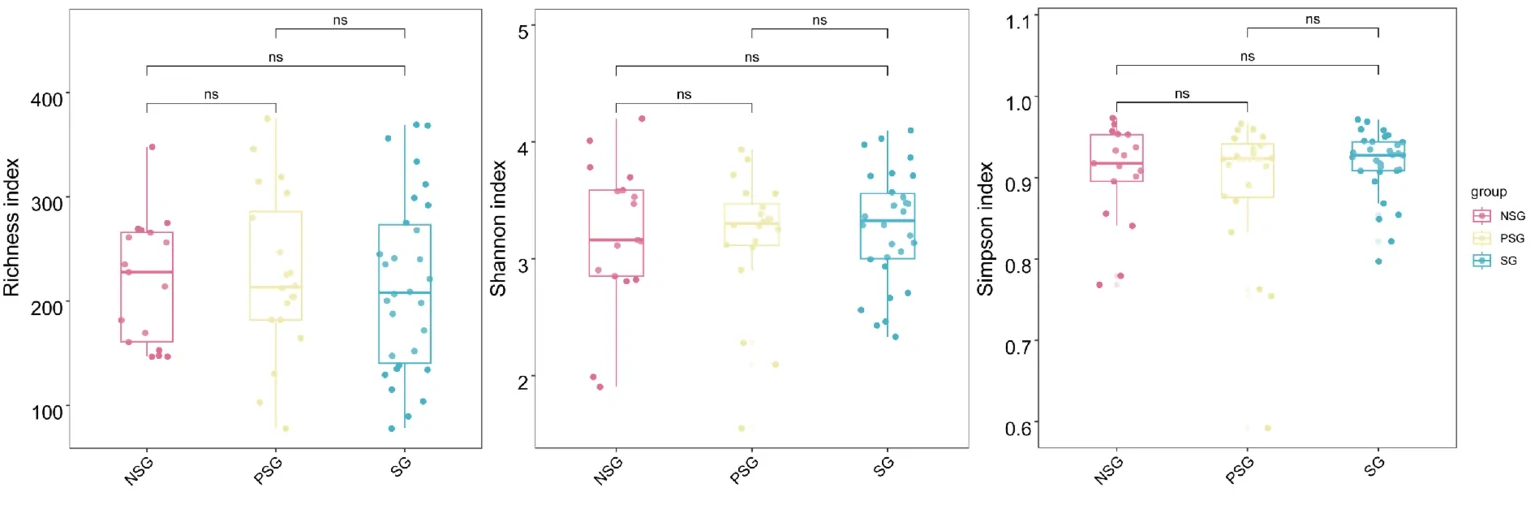
Alpha diversity of the gut microbiome across the three groups. Differences in alpha-diversity indices among the three groups were assessed using the Kruskal–Wallis test.

Beta diversity was evaluated using PCoA to visualize differences in overall microbial community structure among the three groups. As shown in Figure 5, substantial overlap was observed among the NSG, PSG, and SG groups, with no clear group-specific clustering or separation. Consistently, PERMANOVA showed that group membership explained approximately 3% of the variation in overall microbial community structure (*R*^2^ = 0.03), and the overall group effect was not statistically significant (*p* = 0.226).

**Figure 5:**
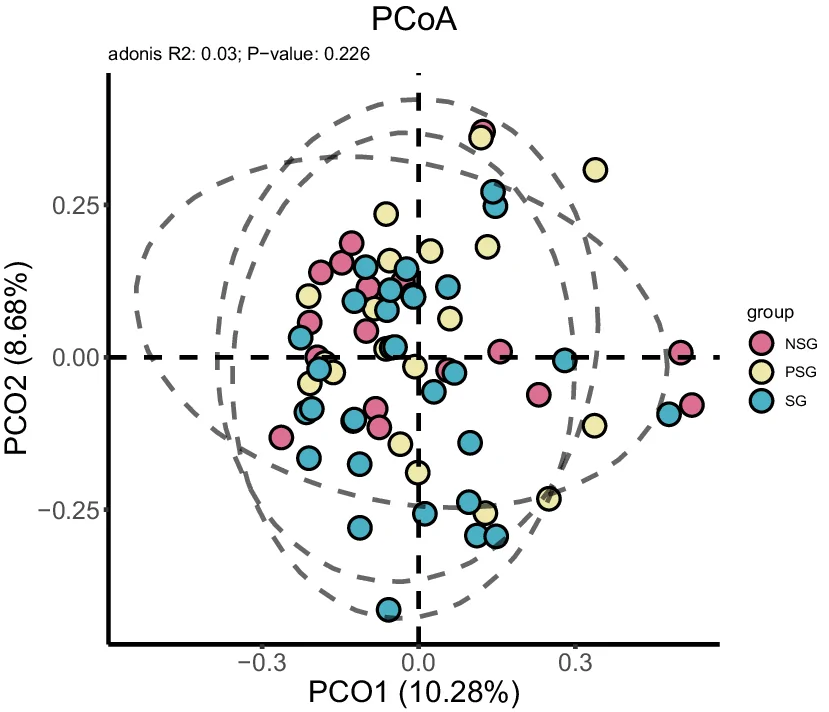
Principal coordinates analysis of gut microbiome composition across the three groups. PCoA was performed based on Bray–Curtis dissimilarities, and differences in overall microbial community composition among the three groups were assessed using PERMANOVA.

#### 3.4.3 Taxonomic composition and differential abundance analysis

##### 3.4.3.1 Taxonomic composition of the gut microbiome at the phylum and genus levels

The taxonomic composition of the gut microbiome was descriptively characterized at the phylum and genus levels using relative abundance profiles (Figure 6). At the phylum level, Bacteroidetes, Firmicutes, Proteobacteria, and Actinobacteria were the predominant phyla across the NSG, PSG, and SG groups (Figure 6A).

**Figure 6:**
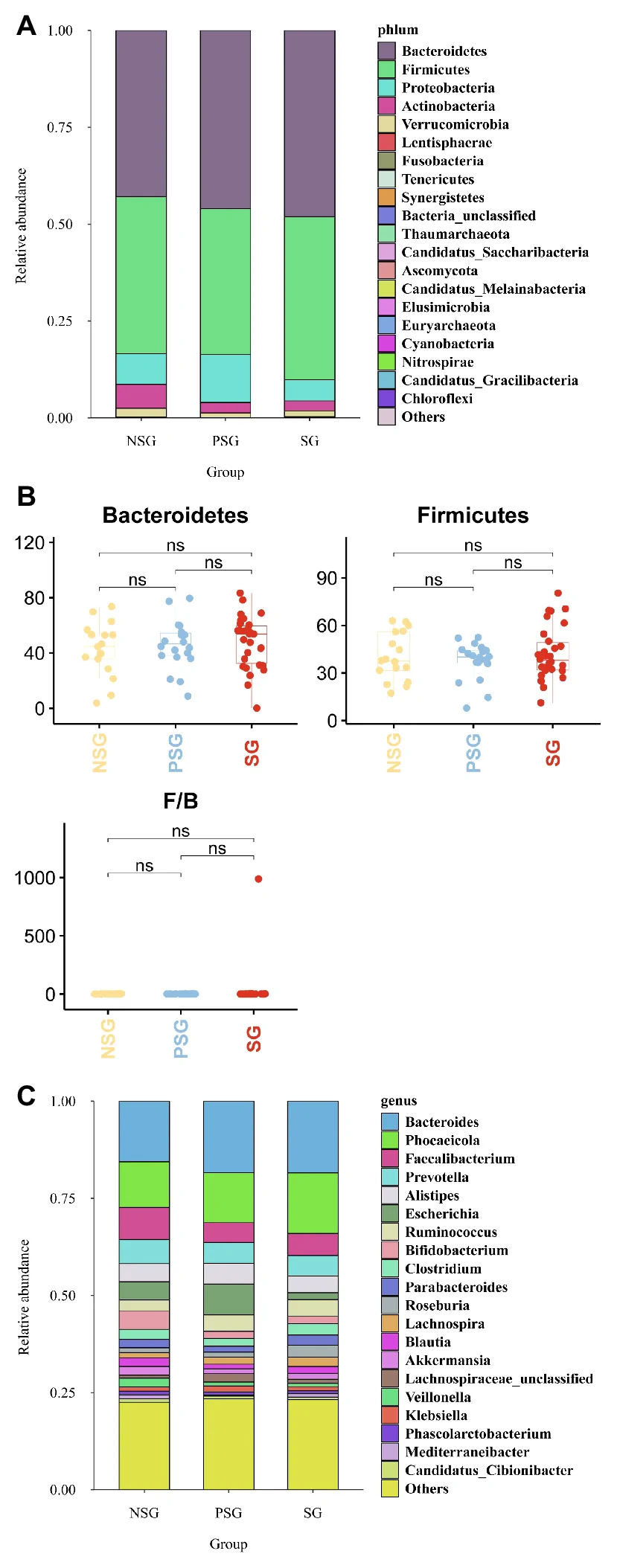
Taxonomic composition of the gut microbiome across the three groups. **(A)** Relative abundance of gut microbiota at the phylum level. **(B)** Firmicutes/Bacteroidetes (F/B) ratio across the three groups. **(C)** Relative abundance of gut microbiota at the genus level.

The Firmicutes/Bacteroidetes (F/B) ratio did not differ significantly among the three groups in the unadjusted analysis (*p* > 0.05; Figure 6B).

At the genus level, *Bacteroides*, *Phocaeicola*, *Faecalibacterium*, *Prevotella*, and *Alistipes* were among the predominant genera across the three groups (Figure 6C). These relative abundance profiles were used to provide a descriptive overview of the taxonomic composition, whereas differential taxonomic abundance was further evaluated using covariate-adjusted MaAsLin2 analysis.

##### 3.4.3.2 Differential abundance analysis

Differential abundance analysis at the genus level was performed using MaAsLin2 with adjustment for age and BMI (Figure 7). In the PSG versus NSG comparison, *Ruminococcus*, *Porphyromonas*, and *Fenollaria* showed nominal positive associations with the PSG group, whereas *Turicibacter* and *Raoultibacter* showed nominal negative associations (*p* < 0.05; Figure 7A). In the SG versus NSG comparison, *Flavonifractor*, *Lachnoclostridium*, *Meridimonas*, and *Actinomyces* showed nominal positive associations with the SG group, whereas *Frisingicoccus* showed a nominal negative association (*p* < 0.05; Figure 7B). In the SG versus PSG comparison, *Enterobacter*, *Tyzzerella*, and *Mediterraneibacter* showed nominal positive associations with the SG group, whereas *Porphyromonas* showed a nominal negative association (*p* < 0.05; Figure 7C).

**Figure 7:**
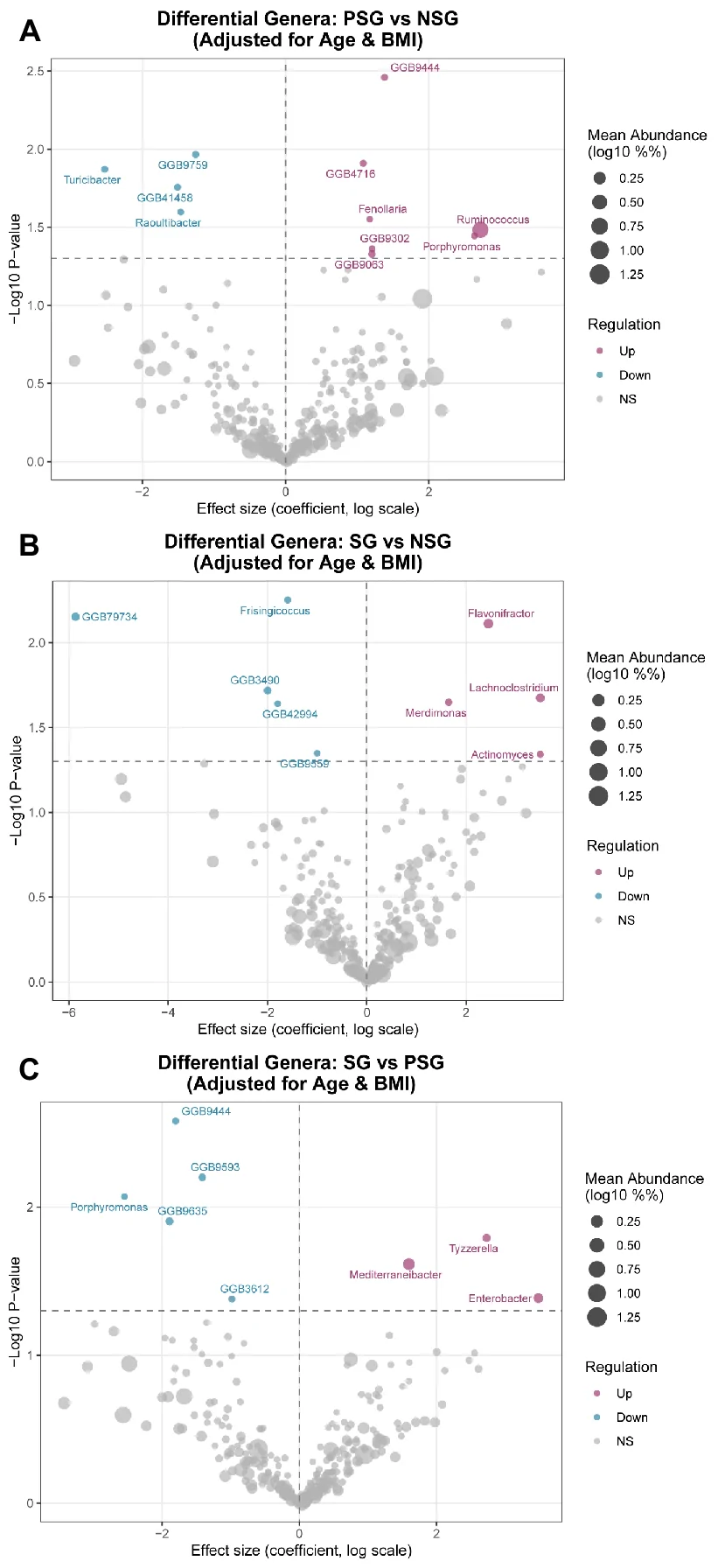
Covariate-adjusted genus-level differential abundance analysis using MaAsLin2. Differential abundance analysis was performed using MaAsLin2 with adjustment for age and body mass index (BMI). **(A)** PSG versus NSG; **(B)** SG versus NSG; and **(C)** SG versus PSG. The x-axis represents the MaAsLin2 regression coefficient (*β*), and the y-axis represents −log10 of the raw *p*-value. Positive and negative *β* coefficients indicate positive and negative associations, respectively, with the first group relative to the reference group. The horizontal dashed line indicates nominal *p* = 0.05. Point size represents mean relative abundance. Colored taxa indicate nominal associations with raw *p* < 0.05, whereas gray taxa were not nominally significant. No genus remained statistically significant after BH-FDR correction (all *q* ≥ 0.05).

However, none of these associations remained statistically significant after Benjamini–Hochberg false discovery rate (BH-FDR) correction(all *q* ≥ 0.05). Therefore, these genus-level associations were considered exploratory and were interpreted cautiously.

### 3.5 Targeted amino acid and kynurenine pathway metabolite profiles

Targeted amino acid profiling was performed to examine differences in circulating amino acid concentrations among the three groups (Figure 8). After adjustment for age and BMI, no statistically significant differences were observed in pairwise comparisons of the measured amino acids among the NSG, PSG, and SG groups (all *p* > 0.05). Although several amino acids showed numerical differences across groups, these differences did not reach statistical significance after covariate adjustment.

**Figure 8:**
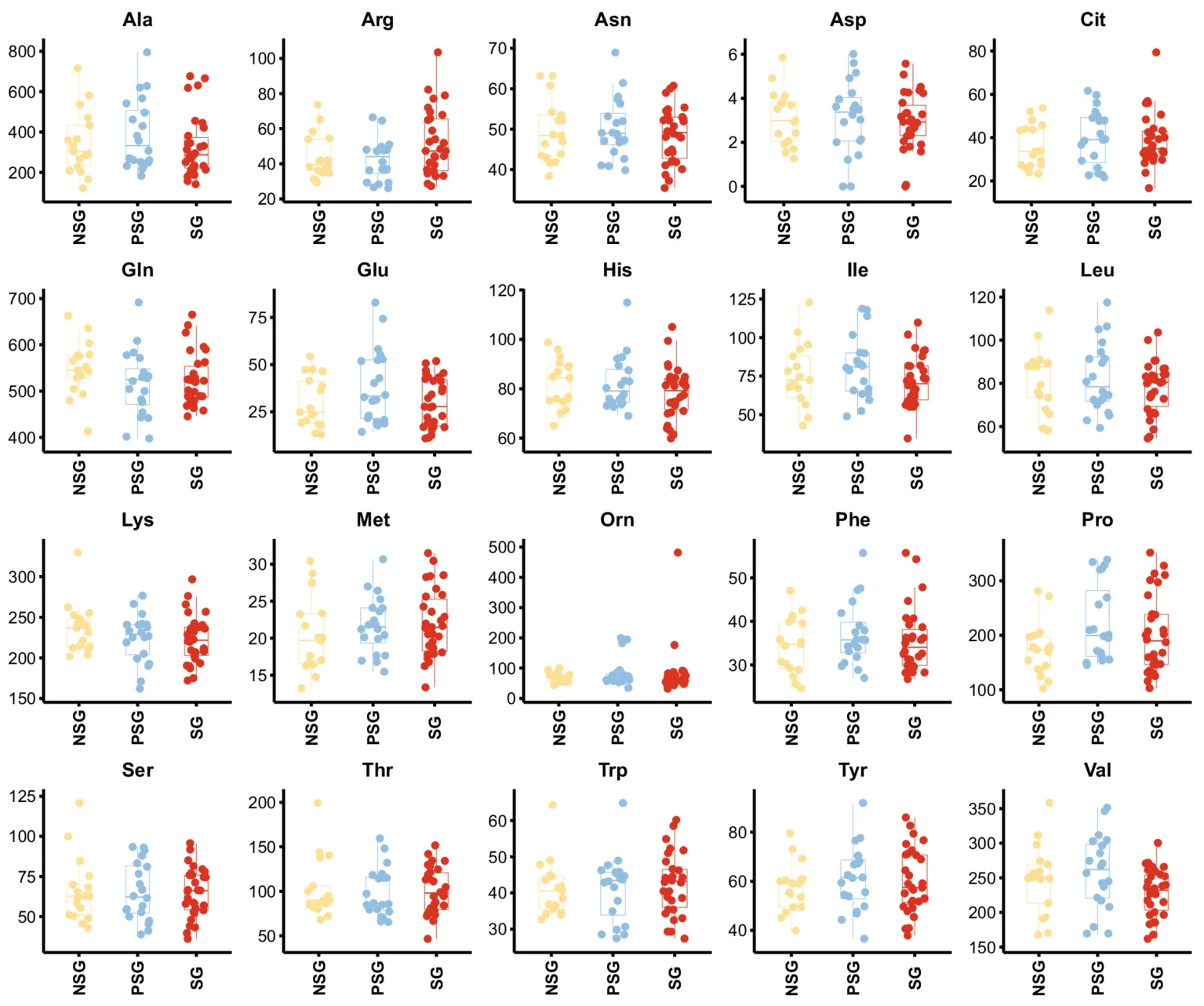
Serum amino acid profiles across the three sarcopenia groups. Individual data points and boxplots show the observed distributions of serum amino acid concentrations in the non-sarcopenia (NSG), possible sarcopenia (PSG), and sarcopenia (SG) groups. The amino acids analyzed included alanine (Ala), arginine (Arg), asparagine (Asn), aspartic acid (Asp), citrulline (Cit), glutamine (Gln), glutamic acid (Glu), histidine (His), isoleucine (Ile), leucine (Leu), lysine (Lys), methionine (Met), ornithine (Orn), phenylalanine (Phe), proline (Pro), serine (Ser), threonine (Thr), tryptophan (Trp), tyrosine (Tyr), and valine (Val). Between-group differences were evaluated using linear regression models adjusted for age and body mass index (BMI).

Serum KYN and Trp concentrations and the KYN/Trp ratio across the three groups are shown in Figure 9. After adjustment for age and BMI, serum KYN concentrations were significantly higher in the SG group than in the NSG group (adjusted mean difference for NSG vs. SG = −339.08, 95% CI: −595.97 to −82.19; *p* = 0.011). No significant differences in KYN concentrations were observed between the NSG and PSG groups (*p* = 0.170) or between the PSG and SG groups (*p* = 0.255).

**Figure 9:**
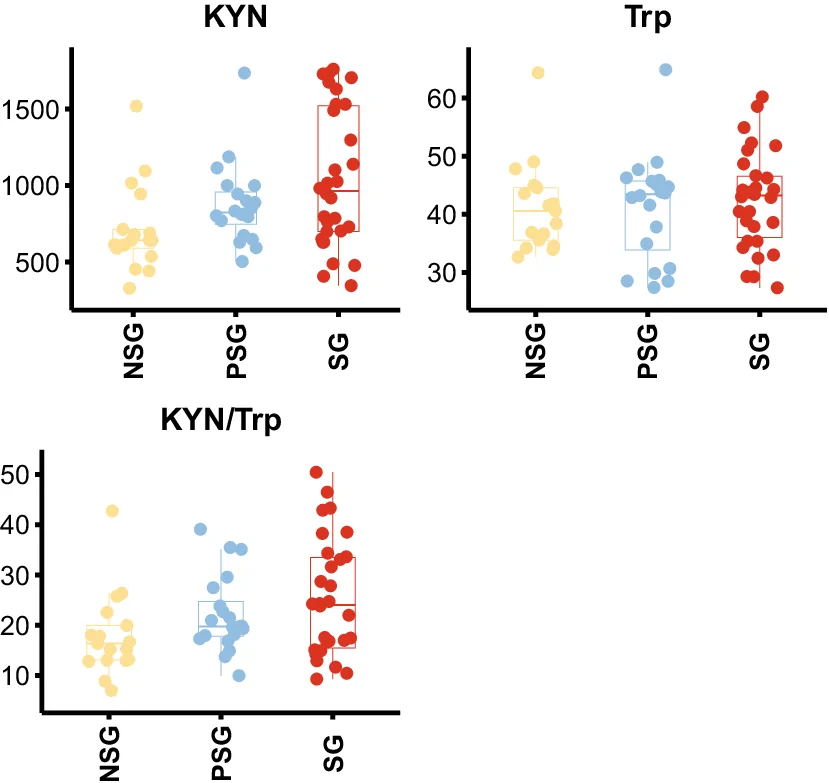
Serum kynurenine (KYN), tryptophan (Trp), and KYN/Trp ratio across the three sarcopenia groups. Individual data points and boxplots show the observed distributions of serum KYN, Trp, and the KYN/Trp ratio in the NSG, PSG, and SG groups. Between-group differences were evaluated using linear regression models adjusted for age and body mass index (BMI).

Trp concentrations did not differ significantly in any of the three pairwise comparisons (all *p* > 0.05). The KYN/Trp ratio was significantly higher in the SG group than in the NSG group after adjustment for age and BMI (adjusted mean difference for NSG vs. SG = −7.03, 95% CI: −13.84 to −0.23; *p* = 0.043), whereas the NSG–PSG and PSG–SG comparisons were not statistically significant (*p* = 0.126 and *p* = 0.686, respectively). Complete covariate-adjusted pairwise comparison results are provided in Supplementary Table S2.

### 3.6 Correlation analysis among gut microbiota, kynurenine pathway metabolites, circulating biomarkers, and physical performance

Correlation analysis revealed associations among microbial genera, kynurenine-related metabolites, circulating biomarkers, and measures of muscle and physical performance (Figure 10). Candidate genera were selected based on nominal associations (*p* < 0.05) identified in age- and BMI-adjusted MaAsLin2 analysis. Because none of these genera remained significant after BH-FDR correction in the differential abundance analysis, the microbiota-related correlation analyses were considered exploratory. *p* values from the correlation analyses were adjusted using the Benjamini–Hochberg false discovery rate procedure.

**Figure 10:**
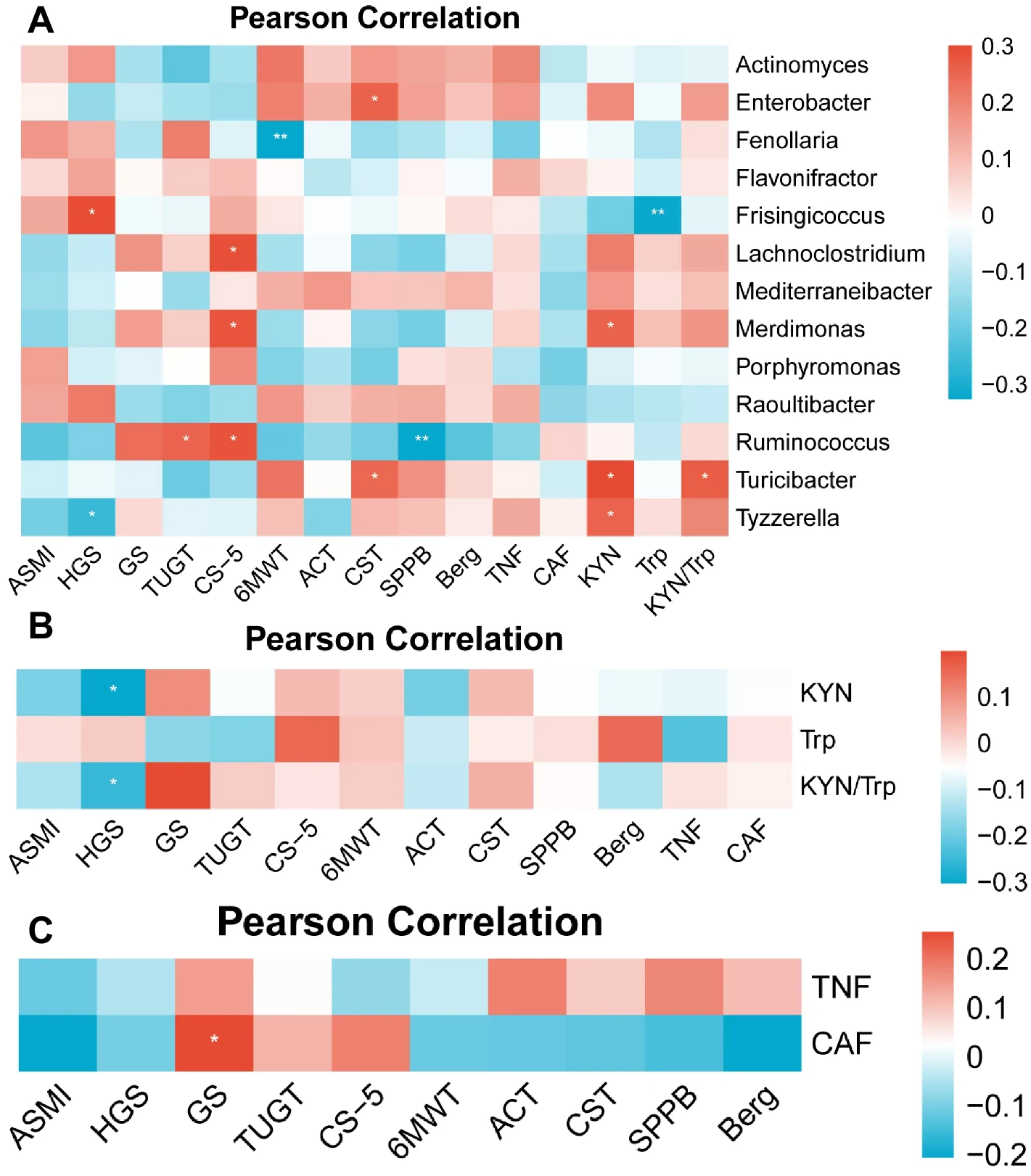
Correlation patterns among gut microbial genera, kynurenine-related metabolites, circulating biomarkers, and muscle and physical performance measures. **(A)** Correlations between candidate gut microbial genera and muscle and physical performance measures, circulating biomarkers, and kynurenine-related metabolites. Candidate genera were selected based on nominal associations (*p* < 0.05) in the age- and BMI-adjusted MaAsLin2 analysis; none remained significant after BH-FDR correction in the differential abundance analysis. **(B)** Correlations of kynurenine (KYN), tryptophan (Trp), and the KYN/Trp ratio with muscle and physical performance measures and circulating biomarkers. **(C)** Correlations of TNF-α and C-terminal agrin fragment (CAF) with muscle and physical performance measures. Correlations were assessed using Pearson’s correlation coefficients based on individual-level observed measurements. Colors represent Pearson’s correlation coefficients (*r*), with red indicating positive and blue indicating negative correlations. *P* values were adjusted using the Benjamini–Hochberg false discovery rate procedure. *q* < 0.05; *q < 0.01.

Among the candidate microbial genera (Figure 10A), several correlations remained significant after BH-FDR correction. *Fenollaria* was negatively correlated with GS, whereas *Frisingicoccus* was positively correlated with HGS and negatively correlated with Trp. *Ruminococcus* was positively correlated with TUGT and CS-5 and negatively correlated with SPPB. *Turicibacter* was positively correlated with CST, KYN, and the KYN/Trp ratio, whereas *Tyzzerella* was negatively correlated with HGS and positively correlated with KYN. Positive correlations were also observed between *Lachnoclostridium* and *Meridimonas* and CS-5, and between *Enterobacter* and CST.

For kynurenine-related metabolites (Figure 10B), both KYN and the KYN/Trp ratio were negatively correlated with HGS. No other correlations between these metabolites and the evaluated muscle or physical performance measures remained significant after correction.

Regarding circulating biomarkers (Figure 10C), CAF was positively correlated with GS, whereas no significant correlations were observed between TNF-α and the evaluated muscle or physical performance measures.

## 4 Discussion

This study investigated differences in muscle characteristics, physical performance, circulating biomarkers, gut microbiota, and amino acid and kynurenine pathway metabolites among older adults classified as NSG, PSG, and SG. By integrating clinical phenotypes with microbial and metabolic measures, the study aimed to explore potential associations among gut microbial alterations, metabolic characteristics, and muscle-related phenotypes across sarcopenia groups. Given the cross-sectional design, the findings should be interpreted as associations rather than evidence of temporal or causal relationships.

### 4.1 Differences in muscle characteristics and physical performance across sarcopenia groups

The present study showed differences in muscle mass and multiple measures of physical performance among the three sarcopenia groups. ASMI was significantly lower in the SG group than in the NSG and PSG groups, whereas no significant difference was observed between the NSG and PSG groups. In contrast, several physical performance measures, including GS, TUGT, CS-5, 6MWT, ACT, CST, SPPB, and Berg Balance Scale scores, differed significantly among the groups. HGS did not differ significantly, although numerically lower values were observed in the PSG and SG groups. These findings suggest that muscle mass, strength, and physical performance do not necessarily exhibit parallel patterns across sarcopenia categories. This is consistent with the clinical concept of “possible sarcopenia” proposed by the AWGS, which emphasizes early identification based on reduced muscle strength and/or physical performance (Cruz-Jentoft et al., 2019; Phu et al., 2020). However, given the cross-sectional design, these findings cannot establish a temporal sequence between functional impairment and loss of muscle mass.

Physical performance also did not show a uniformly stepwise pattern across the NSG, PSG, and SG groups, with the PSG group performing worse than the SG group in TUGT and 6MWT. This non-monotonic pattern may reflect the heterogeneity and multidimensional nature of physical performance. TUGT and 6MWT integrate mobility, balance, endurance, and overall physiological reserve rather than reflecting muscle mass or strength alone (Exter et al., 2024). Moreover, individuals classified as having possible sarcopenia may exhibit substantial impairment in specific functional domains despite not meeting the criterion for low muscle mass. Thus, the poorer TUGT and 6MWT performance in the PSG group likely reflects heterogeneity in functional phenotypes rather than greater disease severity than in established sarcopenia.

Neuromuscular alterations may represent one of several biological factors contributing to these functional differences. Age-related changes in NMJ structure and function can affect motor unit recruitment, muscle activation, and coordinated contraction (Kwon and Yoon, 2017). Nevertheless, physical performance is influenced by multiple physiological systems, and clinical measures such as SPPB and TUGT cannot directly establish NMJ structural alterations (Exter et al., 2024). Therefore, the observed functional differences should not be interpreted as direct evidence of NMJ degeneration.

Circulating CAF showed numerically higher concentrations across the NSG, PSG, and SG groups, consistent in direction with previous reports of elevated CAF in sarcopenia (Fatima et al., 2025; Kupriyanova et al., 2025). However, after adjustment for age and BMI, neither the overall group effect nor pairwise comparisons remained statistically significant. This attenuation may partly reflect the close relationship between sarcopenia, biological aging, and age-related NMJ remodeling, particularly because the PSG and SG groups were older than the NSG group. Nevertheless, the observed effect size (partial *η*^2^ = 0.096) suggests that the relationship between CAF and sarcopenia-related phenotypes warrants further investigation in larger, age-matched cohorts. Overall, CAF may reflect neuromuscular alterations that are partly intertwined with biological aging and should therefore be interpreted cautiously as a potential biomarker of sarcopenia in older adults.

### 4.2 Gut microbiota characteristics across sarcopenia groups

The present study found no significant differences in alpha diversity, beta diversity, or the Firmicutes/Bacteroidetes (F/B) ratio among the NSG, PSG, and SG groups. PCoA showed substantial overlap, with the sarcopenia group explaining only a small proportion of overall microbial variation (PERMANOVA, *R*^2^ = 0.03, *p* = 0.226). These findings suggest that sarcopenia status was not associated with pronounced alterations in overall gut microbial diversity or community composition in this cohort. Previous studies have similarly suggested that aging- and disease-related microbial alterations may involve changes in specific taxa rather than marked shifts in overall diversity (Chong et al., 2025).

At the genus level, none of the associations identified by covariate-adjusted MaAsLin2 remained significant after BH-FDR correction. Nevertheless, several nominal associations were considered exploratory, including higher *Porphyromonas* in the PSG group relative to the NSG group and higher *Enterobacter* in the SG group relative to the PSG group. Descriptive profiles also suggested variation in *Faecalibacterium*, although this was not statistically confirmed. These observations may be biologically relevant because microbial metabolites such as SCFAs contribute to intestinal barrier integrity and immune homeostasis (Peng et al., 2009; Sokol et al., 2008), whereas products of Gram-negative bacteria, including lipopolysaccharide (LPS), can activate TLR4/NF-κB signaling and promote inflammatory responses (Zhang et al., 2008; Qiu et al., 2021). *Enterobacter*, a member of the Gram-negative Enterobacteriaceae, may therefore be of potential interest in this context, consistent with previous links between gut-derived Gram-negative bacteria, LPS-related signaling, and metabolic inflammation (Iqbal et al., 2025; Watanabe et al., 2021).

However, circulating LPS, intestinal permeability, and microbial functional activity were not assessed, and TNF-α did not differ significantly among the groups. Circulating TNF-α also shows substantial interindividual variability, while chronic low-grade inflammation may involve coordinated changes across multiple inflammatory mediators rather than elevation of a single cytokine (Cheng et al., 2025; Du et al., 2022; Ibrahim et al., 2024). Together with the absence of FDR-significant genus-level associations, these limitations preclude conclusions regarding activation of an LPS–TLR4–NF-κB pathway or a causal role of specific taxa in sarcopenia. The observed taxonomic patterns should therefore be considered hypothesis-generating and require validation in larger longitudinal and mechanistic studies.

### 4.3 Alterations in tryptophan metabolism across sarcopenia groups

The present study found significantly higher serum KYN concentrations and KYN/Trp ratios in the SG group than in the NSG group after adjustment for age and BMI, whereas Trp concentrations did not differ significantly. These findings indicate altered kynurenine-related tryptophan metabolism associated with established sarcopenia.

The conversion of tryptophan to KYN is regulated by indoleamine 2,3-dioxygenase (IDO) and tryptophan 2,3-dioxygenase (TDO), with IDO1 being responsive to inflammatory signaling (Ballesteros et al., 2023; Tero-Vescan et al., 2025). Although the KYN/Trp ratio is commonly used as an indirect index of tryptophan degradation through the kynurenine pathway, circulating metabolite concentrations are also influenced by production, utilization, transport, and clearance. Because IDO/TDO activity was not directly assessed, the observed increases in KYN and KYN/Trp indicate altered kynurenine-related metabolism but do not establish increased pathway flux. Moreover, KYN is further metabolized into metabolites with distinct biological properties, including kynurenic acid (KYNA), 3-hydroxykynurenine (3-HK), anthranilic acid (AA), xanthurenic acid (XA), and quinolinic acid (QUIN), which have been implicated in oxidative, inflammatory, and neuroactive processes (Chen et al., 2021; Xu et al., 2024). As these downstream metabolites were not measured, the direction of downstream kynurenine metabolism cannot be determined.

Importantly, kynurenine metabolism represents only one branch of tryptophan metabolism. Gut microorganisms also metabolize tryptophan through indole-producing pathways, generating metabolites including indole-3-acetic acid (IAA), indole-3-lactic acid (ILA), indole-3-propionic acid (IPA), and indole-3-aldehyde (IAld) (Roager and Licht, 2018). These metabolites contribute to host–microbiome communication and can modulate intestinal barrier integrity and immune homeostasis through signaling pathways involving AhR, PXR, and IL-22 (Roager and Licht, 2018; Venkatesh et al., 2014; Zelante et al., 2013). Host kynurenine and microbial indole metabolism therefore represent distinct but interconnected components of tryptophan metabolism (Agus et al., 2018; Xue et al., 2023). However, because microbial indole metabolites were not quantified, the present study cannot determine whether microbial tryptophan metabolism differed among the sarcopenia groups or contributed to the observed KYN-related phenotype.

Alterations in tryptophan metabolism may also be relevant to neuromuscular health. Age-related NMJ remodeling is associated with oxidative stress, mitochondrial dysfunction, inflammation, and denervation (Gonzalez-Freire et al., 2014; Rudolf et al., 2016). Microbial indole metabolites may indirectly influence this physiological environment through effects on intestinal barrier and immune homeostasis (Krishnamurthy et al., 2023), whereas KYN and its downstream metabolites have been implicated in oxidative stress, neuronal injury, and skeletal muscle dysfunction (Kaiser et al., 2019). Altered kynurenine metabolism has also been associated with frailty and age-related muscle and functional decline (Chung et al., 2023). Nevertheless, direct evidence linking either microbial indole metabolism or circulating KYN to NMJ remodeling in sarcopenia remains limited, and such mechanisms were not directly assessed in the present study.

Notably, higher KYN and KYN/Trp were observed despite the absence of pronounced differences in overall gut microbial community structure or FDR-significant genus-level associations. Thus, microbial taxonomic composition and circulating tryptophan-related metabolic phenotypes may not necessarily vary in parallel. Overall, the present findings support an association between established sarcopenia and altered kynurenine-related metabolism but do not establish kynurenine pathway activation or a causal microbiota–tryptophan metabolism–NMJ pathway. Future studies integrating microbial indole metabolites, downstream kynurenine metabolites, IDO/TDO activity, microbiome functional profiling, and direct assessments of NMJ structure and function are needed to clarify these relationships.

### 4.4 Associations among gut microbiota, kynurenine-related metabolism, and physical function

Correlation analyses further revealed several associations among candidate microbial genera, kynurenine-related metabolites, circulating biomarkers, and physical performance measures. Although these analyses were exploratory, several associations remained significant after BH-FDR correction and showed potentially relevant patterns across microbial, metabolic, and functional domains.

Among the candidate microbial genera, several taxa were associated with muscle strength, physical performance, and kynurenine-related metabolites. These exploratory associations may reflect broader host–microbiome interactions in tryptophan metabolism rather than direct regulation of circulating KYN by individual genera. Gut microorganisms metabolize tryptophan into indole derivatives that participate in host–microbiome signaling and can influence intestinal barrier integrity, AhR-dependent immune responses, and inflammatory homeostasis (Agus et al., 2018; Gonzalez-Freire et al., 2014; Roager and Licht, 2018; Venkatesh et al., 2014; Xue et al., 2023; Zelante et al., 2013). Such microbial metabolic activity may therefore contribute to the host environment in which tryptophan metabolism occurs. However, microbial indole metabolites and tryptophan-metabolism-related microbial functional genes were not measured in the present study, and the mechanisms underlying these associations remain uncertain.

Skeletal muscle actively participates in kynurenine metabolism through kynurenine aminotransferases (KATs), which convert KYN to kynurenic acid (KYNA). Experimental studies have shown that skeletal muscle PGC-1α1 enhances KAT expression and promotes peripheral KYN-to-KYNA conversion, linking muscle metabolic capacity to systemic kynurenine metabolism (Agudelo et al., 2014). Altered kynurenine metabolism has also been associated with age-related muscle dysfunction and frailty (Hinkley et al., 2023; Jang et al., 2020; Kaiser et al., 2019). Thus, the observed associations between higher KYN-related measures and lower muscle strength may reflect bidirectional interactions between systemic kynurenine metabolism and skeletal muscle physiology. However, given the cross-sectional design and the absence of downstream kynurenine metabolites or direct measurements of IDO/TDO activity, the direction and underlying metabolic pathways cannot be determined.

CAF was positively correlated with GS, indicating that higher CAF levels were associated with poorer walking performance. This finding is consistent with previous studies linking elevated circulating CAF, a marker associated with agrin cleavage and NMJ remodeling, with impaired muscle strength and physical performance (Fatima et al., 2025; Landi et al., 2016; Marzetti et al., 2014). Age-related NMJ remodeling involves progressive denervation–reinnervation imbalance and is influenced by mitochondrial dysfunction and oxidative stress, which may contribute to impaired neuromuscular transmission and physical function (Anagnostou and Hepple, 2020; Chai et al., 2026). Thus, the observed CAF–walking association may reflect broader age-related neuromuscular alterations. Nevertheless, CAF is an indirect circulating marker, and this association does not establish that NMJ remodeling caused the observed impairment in walking performance.

Taken together, these findings are consistent with potential interactions among gut microbial characteristics, host–microbial tryptophan metabolism, skeletal muscle metabolism, and neuromuscular function. Rather than supporting a unidirectional microbiota–kynurenine–NMJ pathway, the observed correlations may reflect a bidirectional network involving microbial, systemic metabolic, muscular, and neuromuscular processes. However, because the candidate microbial taxa were derived from nominal MaAsLin2 associations that did not survive FDR correction and the analyses were cross-sectional, these findings should be considered hypothesis-generating and do not establish causal relationships among these biological domains.

### 4.5 Limitations

Several limitations should be considered. First, the cross-sectional design precludes causal or temporal inference among gut microbial characteristics, tryptophan metabolism, neuromuscular biomarkers, and sarcopenia-related phenotypes. Second, the relatively small and unequal group sizes may have limited statistical power, particularly for microbiome and biomarker analyses. Although relevant analyses were adjusted for age and BMI, residual confounding cannot be excluded. Moreover, none of the genus-level associations remained significant after BH-FDR correction and should therefore be considered exploratory.

Third, tryptophan metabolism was incompletely characterized. Downstream kynurenine metabolites (KYNA, 3-HK, AA, XA, and QUIN), IDO/TDO activity, and microbiota-derived indole metabolites such as IAA and IPA were not assessed. Thus, the present findings cannot establish kynurenine pathway flux or characterize potential interactions between host kynurenine and microbial indole metabolism. Finally, CAF provides an indirect circulating measure related to NMJ remodeling, whereas direct assessments of NMJ structure or function were not performed. Future longitudinal studies integrating comprehensive tryptophan metabolite profiling, microbiome functional analyses, and direct NMJ assessments are needed to clarify these relationships.

## 5 Conclusion

The present study identified distinct clinical and metabolic characteristics across sarcopenia groups. Muscle mass, strength, and physical performance did not exhibit parallel patterns, highlighting the heterogeneity of sarcopenia-related phenotypes. Overall gut microbial diversity and community composition showed no pronounced between-group differences, whereas circulating KYN and the KYN/Trp ratio were significantly higher in established sarcopenia after adjustment for age and BMI, supporting an association between sarcopenia and altered kynurenine-related metabolism. CAF differences were attenuated after covariate adjustment, suggesting that its relationship with sarcopenia may be partly intertwined with aging.

Exploratory correlations further revealed associations across microbial, kynurenine-related metabolic, muscular, and neuromuscular domains. Collectively, these findings are consistent with a potentially interconnected and bidirectional network involving gut microbial characteristics, tryptophan metabolism, skeletal muscle, and neuromuscular function, rather than a unidirectional causal microbiota–kynurenine–NMJ pathway. Longitudinal and mechanistic studies are required to clarify these relationships.

## Data Availability

The datasets generated and/or analyzed during the current study are available from the corresponding authors upon reasonable request.
