# Supplementary material for: Gut microbial characteristics and kynurenine-related metabolism across sarcopenia groups: associations with neuromuscular and physical function
Source: Front Aging Neurosci. 2026 Sep 25;18:1883616. doi: 10.3389/fnagi.2026.1883616 (PMC13649696; doi:10.3389/fnagi.2026.1883616)
Supplement: Supplementary file 1 [file Data_Sheet_1.DOCX]

Supplementary Material

**Supplementary Table 1.** Adjusted between-group differences in CAF and TNF-α after controlling for age, sex, and BMI.

| **Feature** | **Comparison** | **Adjusted estimate** | **SE** | **95% CI** | **Adjusted *P*** | **Standardized effect size** | **Overall partial η^2^** |
| --- | --- | --- | --- | --- | --- | --- | --- |
| CAF | NSG vs PSG | 0.006 | 0.029 | −0.064 to 0.076 | 0.976 | 0.078 | 0.096 |
|  | NSG vs SG | −0.054 | 0.027 | −0.120 to 0.011 | 0.124 | −0.685 |  |
|  | PSG vs SG | −0.061 | 0.028 | −0.127 to 0.006 | 0.080 | −0.763 |  |
| TNF-α | NSG vs PSG | 8.261 | 30.975 | −66.118 to 82.640 | 0.962 | 0.098 | 0.001 |
|  | NSG vs SG | 8.436 | 29.017 | −61.242 to 78.115 | 0.955 | 0.100 |  |
|  | PSG vs SG | 0.175 | 29.262 | −70.090 to 70.441 | 1.000 | 0.002 |  |

Multivariable models were adjusted for age, sex, and body mass index (BMI). Adjusted between-group contrasts were estimated using estimated marginal means and are presented as adjusted estimates with standard errors (SEs), 95% confidence intervals (CIs), multiplicity-adjusted *P* values, and standardized effect sizes. Partial η^2^ represents the overall effect size of group after covariate adjustment. NSG, non-sarcopenia group; PSG, possible sarcopenia group; SG, sarcopenia group; CAF, C-terminal agrin fragment; TNF-α, tumor necrosis factor-α.

**Supplementary Table 2.** Age- and BMI-adjusted pairwise comparisons of serum kynurenine pathway-related metabolites among the three groups.

| **Metabolite** | **Comparison** | **Adjusted mean difference** | **SE** | **95% CI** | ***t* value** | ***P* value** |
| --- | --- | --- | --- | --- | --- | --- |
| KYN | NSG vs PSG | −190.31 | 137.18 | −464.54 to 83.91 | −1.39 | 0.170 |
| KYN | NSG vs SG | −339.08 | 128.51 | −595.97 to −82.19 | −2.64 | **0.011** |
| KYN | PSG vs SG | −148.77 | 129.60 | −407.83 to 110.29 | −1.15 | 0.255 |
| Trp | NSG vs PSG | 1.23 | 3.13 | −5.02 to 7.49 | 0.39 | 0.695 |
| Trp | NSG vs SG | −2.35 | 2.93 | −8.21 to 3.51 | −0.80 | 0.426 |
| Trp | PSG vs SG | −3.58 | 2.96 | −9.49 to 2.33 | −1.21 | 0.230 |
| KYN/Trp | NSG vs PSG | −5.64 | 3.63 | −12.90 to 1.63 | −1.55 | 0.126 |
| KYN/Trp | NSG vs SG | −7.03 | 3.40 | −13.84 to −0.23 | −2.07 | **0.043** |
| KYN/Trp | PSG vs SG | −1.39 | 3.43 | −8.26 to 5.47 | −0.41 | 0.686 |

Data represent pairwise contrasts derived from linear regression models adjusted for age and body mass index (BMI). Estimated marginal means and pairwise contrasts were obtained using the emmeans package. Adjusted mean differences represent the first group minus the second group in each comparison. Negative estimates therefore indicate lower values in the first group relative to the second group. P values are two-sided and were not adjusted for multiple pairwise comparisons.
